# Long-Term Body Composition Trajectories After Bariatric Surgery: A 5-Year Comparative Study of Biliopancreatic Diversion, Roux-en-Y Gastric Bypass, and Sleeve Gastrectomy

**DOI:** 10.3390/jcm15062354

**Published:** 2026-03-19

**Authors:** María Antequera-González, Elena González Arnáiz, Diana G. Ariadel-Cobo, Diana García Sastre, María López Melgar, Ana Urioste Fondo, M. Carmen Dameto Pons, María Casado Rodríguez, Jesús Manuel Silva Fernández, Luis González-Herráez García, María D. Ballesteros-Pomar

**Affiliations:** 1 Department of Endocrinology and Nutrition, Complejo Asistencial Universitario de León (CAULE), 24008 Leon, Spain; egonzalezar@saludcastillayleon.es (E.G.A.); dgariadel@saludcastillayleon.es (D.G.A.-C.); dgarciasastre@saludcastillayleon.es (D.G.S.); mlopezmel@saludcastillayleon.es (M.L.M.); amurioste@saludcastillayleon.es (A.U.F.); mcdameto@saludcastillayleon.es (M.C.D.P.); mcasadorod@saludcastillayleon.es (M.C.R.); dballesteros@saludcastillayleon.es (M.D.B.-P.); 2 Department of General Surgery, Complejo Asistencial Universitario de León (CAULE), 24008 Leon, Spain; jsilvaf@saludcastillayleon.es (J.M.S.F.); lgonzalez-herraez@saludcastillayleon.es (L.G.-H.G.); 3 Institute of Biomedicine (IBIOMED), University of León, 24071 Leon, Spain

**Keywords:** bariatric surgery, body composition, fat mass, fat-free mass, biliopancreatic diversion, sleeve gastrectomy, roux-en-Y gastric bypass

## Abstract

**Background/Objectives**: Long-term comparative data on body composition (BC) trajectories following different bariatric procedures remain limited, particularly regarding potential muscle preservation after malabsorptive techniques. We aimed to compare 5-year changes in adiposity and muscle mass following biliopancreatic diversion (BPD), Roux-en-Y gastric bypass (RYGB), and sleeve gastrectomy (SG), adjusting for baseline heterogeneity. **Methods**: In this retrospective longitudinal study, 128 patients with severe obesity were followed for 60 months. BC was assessed annually using multi-frequency bioelectrical impedance analysis. Multivariable linear mixed-effects models adjusted for baseline BMI, age, and sex were used to evaluate trajectories of total weight loss (%WL), fat mass loss (%FML), and skeletal muscle mass (SMM). **Results**: BPD demonstrated a significantly superior longitudinal trajectory for %WL (β = 0.124 [95% CI: 0.013–0.235], *p* = 0.028) and %FML (β = 0.288 [95% CI: 0.135–0.440], *p* < 0.001) over 5 years. However, no statistically significant independent differences between techniques were observed at the isolated 60-month endpoint after full adjustment. Although BPD was associated with a higher percentage of muscle mass loss (*p* = 0.019), absolute skeletal and appendicular muscle mass did not differ significantly across procedures. Age emerged as an independent negative predictor of weight and fat loss (*p* < 0.001). **Conclusions**: After rigorous adjustment for baseline characteristics, BPD provides greater long-term adiposity reduction without evidence of disproportionate impairment of absolute muscle mass compared with RYGB or SG. These findings contribute to a more refined understanding of long-term body composition dynamics following bariatric surgery.

## 1. Introduction

Severe obesity is associated with profound alterations in body composition (BC), including excess adiposity and impaired muscle quality, which contribute significantly to long-term cardiometabolic and functional risk [1,2]. While bariatric surgery (BS) is established as the most effective intervention for sustained weight loss, its success should not be evaluated solely by total weight reduction or body mass index (BMI) [3,4]. The quality of weight loss—defined by the ratio of fat mass (FM) reduction to the preservation of fat-free mass (FFM) and skeletal muscle mass (SMM)—is a critical determinant of post-surgical metabolic health and long-term physical independence [5,6].

Despite the widespread use of procedures such as sleeve gastrectomy (SG) and Roux-en-Y gastric bypass (RYGB), there is significant clinical uncertainty regarding the long-term musculoskeletal safety of more aggressive techniques, such as biliopancreatic diversion (BPD) [7,8]. While BPD is renowned for its superior efficacy in fat mass reduction, it remains unclear whether this metabolic advantage comes at the cost of disproportionate muscle wasting over a five-year horizon [9,10]. Most available studies report only short- to mid-term outcomes and frequently lack the necessary multivariable adjustments to account for baseline differences in age and obesity severity, factors that may significantly bias inter-technique comparisons [11,12].

Furthermore, the longitudinal monitoring of BC trajectories faces major methodological challenges, including high attrition rates and baseline clinical heterogeneity [13,14]. These factors can lead to inaccurate estimations of surgical impact if the data are not analyzed through an integrated approach that accounts for individual patient characteristics over time [15,16]. There is a critical need for studies that isolate the true effect of surgical techniques from baseline confounders, such as age and initial BMI, to provide more reliable evidence for clinical decision-making [17,18].

Therefore, the primary objective of this study was to evaluate and compare the 5-year trajectories of body composition changes following BPD, RYGB, and SG in a cohort of patients with severe obesity. Specifically, we sought to determine whether the superior fat mass loss associated with BPD compromises skeletal and appendicular muscle mass after rigorous adjustment for baseline BMI, age, and sex, thereby ensuring a more accurate assessment of the long-term functional and metabolic safety of these surgical interventions.

## 2. Materials and Methods

### 2.1. Study Design and Participants

A retrospective longitudinal study was conducted on a cohort of patients with severe obesity who underwent bariatric surgery at a tertiary referral center between January 2005 and December 2023. Patients were followed over a 5-year period (60 months). Inclusion criteria were defined according to international clinical guidelines for metabolic surgery [15,16]: (1) BMI ≥ 40 kg/m^2^ or ≥ 35 kg/m^2^ with associated comorbidities, (2) age between 18 and 65 years, and (3) availability of complete body composition data at baseline and at least three follow-up time points. Patients undergoing revisional surgery, those with active malignancy, or severe psychiatric disorders were excluded. The study was conducted in accordance with the Declaration of Helsinki and was approved by the Institutional Ethics Committee (Protocol No.: 2104).

### 2.2. Surgical Procedures

Patients were assigned to one of three standardized procedures: BPD, RYGB, or SG. All procedures were performed by a multidisciplinary team following standardized surgical protocols to ensure consistency in limb lengths and procedural technique [17]. The selection of the surgical technique was performed based on the patient’s metabolic profile, baseline BMI, and eating behavior.

### 2.3. Anthropometry and Body Composition Assessment

Anthropometric evaluations were performed at baseline and annually at 12, 24, 36, 48, and 60 months postoperatively. Weight was measured to the nearest 0.1 kg using a calibrated TANITA^®^ (Tokyo, Japan) MC-780A scale; height to the nearest 0.1 cm with a SECA 213 stadiometer, following International Society for the Advancement of Kinanthropometry (ISAK) guidelines (13). Total weight loss percentage (%WL) and specific body composition change percentages (e.g., %FML, %MML) were calculated individually for each subject at each follow-up point relative to their specific baseline values [18].

BC was assessed through multi-frequency TANITA^®^ MC-780A bioimpedance analyzer. To ensure reliability and minimize measurement bias related to hydration sensitivity, patients followed a standardized protocol: an 8 h fast, empty bladder, and avoidance of strenuous exercise 24 h prior to the assessment [9,10]. BIA provided data on FM, FFM, and SMM. Appendicular muscle mass (AMM) was calculated as the sum of lean mass in the extremities. The use of BIA has been validated against dual-energy X-ray absorptiometry (DXA) for monitoring bariatric patients [11,12,19].

### 2.4. Statistical Analysis

To analyze the longitudinal trajectories of body composition and manage the attrition rate (72.7% at 60 months), Multivariable Linear Mixed-Effects Models (LMM) were implemented [20,21,22]. This approach was chosen over repeated-measures ANOVA as it accounts for missing data under the Missing At Random (MAR) assumption, utilizing all available data points to minimize attrition bias. Time was treated as a categorical variable (annual visits). Surgical Technique, Time, and their interaction (Technique × Time) were included as fixed effects. The models were adjusted for baseline BMI, Age, and Sex as fixed-effect covariates to account for initial heterogeneity [17,23]. To model the correlation structure of repeated measures, an unstructured covariance matrix was utilized. Random intercepts per patient were included to account for individual baseline variability. Effect sizes were reported as fixed-effect coefficients (β) with their corresponding 95% Confidence Intervals (95% CI). Analyses were performed using IBM SPSS Statistics, v.28.0 (IBM Corp., Armonk, NY, USA). Statistical visualizations were generated using Python with specialized libraries (Matplotlib and Seaborn) to represent the adjusted trajectories derived from the LMM models. Statistical significance was set at *p* < 0.05.

## 3. Results

### 3.1. Baseline Profile and Attrition Analysis

The study included a total of 128 patients. Baseline clinical characteristics according to the surgical procedure are summarized in Table 1. Significant differences were observed at baseline regarding BMI (BPD: 52.3 ± 8.3 vs. RYGB: 43.6 ± 5.8 vs. SG: 42.7 ± 4.2 kg/m^2^; *p* < 0.001) and age (BPD: 43.1 ± 9.3 vs. RYGB: 46.8 ± 9.1 vs. SG: 48 ± 9.4 years; *p* = 0.024), which justifies the use of adjusted multivariable models for longitudinal comparisons.

A sensitivity analysis was performed to evaluate the impact of the attrition rate at the 60-month follow-up. As shown in Table 2, no statistically significant differences were found between patients who completed the 5-year follow-up (*n* = 36) and those lost to follow-up (*n* = 92) regarding baseline age, weight, or BMI (all *p* > 0.05). These findings suggest that the final sample remains representative of the initial cohort and support the MAR assumption for the longitudinal analysis.

### 3.2. Multivariable Analysis of Longitudinal Trajectories

To account for repeated measures and control for baseline clinical heterogeneity, a multivariable LMM with a random intercept for each subject was employed. The coefficients for the trajectories of %WL and fat mass loss (%FML) are detailed in Table 3.

**Figure 1:**
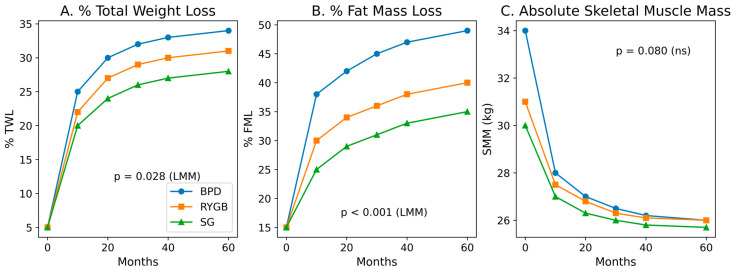
Long-term (5-year) trajectories of body composition following BPD, RYGB, and SG. (**A**) Percentage of total weight loss (%WL). (**B**) Percentage of fat mass loss (%FML). (**C**) Absolute skeletal muscle mass (SMM) in kilograms. Solid lines represent adjusted mean estimates from multivariable linear mixed-effects models (LMM) adjusted for baseline BMI, age, and sex. BPD: Biliopancreatic Diversion; RYGB: Roux-en-Y Gastric Bypass; SG: Sleeve Gastrectomy.

The model identified a significant Time x Technique interaction for the BPD group in both %WL (beta = 0.124 [95% CI: 0.013, 0.235], *p* = 0.028) and %FML (beta = 0.288 [95% CI: 0.135, 0.440], *p* < 0.001). Adjusted trajectories are illustrated in Figure 1 (Panels A and B), showing a sustained metabolic advantage for BPD throughout the 60-month horizon compared to RYGB and SG.

Analysis of covariates confirmed that age was a significant independent negative predictor of weight loss efficacy (beta = −0.420, *p* < 0.001). Additionally, baseline BMI was found to be a significant predictor for %WL trajectories (beta = 0.289 [95% CI: 0.029, 0.548], *p* = 0.029), whereas no such significant effect was observed for %FML (*p* = 0.584). Sex (male) did not significantly influence weight loss or fat mass loss trajectories in this adjusted model (*p* = 0.893 and *p* = 0.137, respectively).

### 3.3. Five-Year Evolution of Body Composition and Muscle Health

The detailed longitudinal evolution is summarized in Table 4 and Table 5. Numerically, the BPD group showed a higher mean percentage of fat mass loss at 60 months (57.9 ± 18.3%) compared to RYGB (39.8 ± 14.3%) and SG (42.0 ± 12.5%).

Regarding musculoskeletal health, no statistically significant differences were observed in SMM (*p* = 0.388) or AMM (*p* = 0.194) across techniques in the adjusted longitudinal models. While the BPD group showed a higher percentage of total muscle mass loss (%MML, 20.6 ± 5.5%, *p* = 0.019), absolute functional muscle mass values remained comparable across groups. All *p*-values reported for longitudinal comparisons were derived from linear mixed-effects models adjusted for baseline BMI, age, and sex. Overall, these findings indicate that inter-technique differences are primarily reflected in longitudinal adiposity trajectories rather than in absolute long-term muscle mass preservation.

## 4. Discussion

BS induces profound and sustained alterations in BC that extend beyond total weight loss, encompassing clinically relevant long-term modifications in FM, FFM, and SMM [1,4]. While total weight reduction remains the primary therapeutic target, contemporary metabolic research increasingly emphasizes the qualitative composition of weight loss as a determinant of long-term cardiometabolic and functional outcomes [2,3,4,5,6]. In this 5-year longitudinal cohort, we implemented multivariable LMM to isolate the independent effect of surgical technique from baseline heterogeneity in BMI and age, variables known to influence postoperative trajectories [23]. Our principal finding is that BPD confers a significantly superior long-term trajectory of adiposity reduction compared with RYGB and SG, without evidence of disproportionate impairment of absolute SMM in adjusted analyses.

### 4.1. Long-Term Adiposity Reduction: Trajectories Versus Isolated Endpoints

After rigorous adjustment, BPD demonstrated a significant Technique × Time interaction for both %WL (*p* = 0.028) and %FML (*p* < 0.001), indicating a sustained metabolic advantage across the 60-month horizon. Importantly, although crude 5-year means numerically favored BPD, statistical differences were attenuated at the isolated 60-month endpoint. This distinction is methodologically and clinically relevant. Long-term comparative cohorts [15,16] and randomized data such as the SLEEVEPASS trial [24] have shown that between-technique differences may narrow over time once baseline imbalances are accounted for. Similarly, Ceriani et al. [17] reported differential trajectories in weight and composition change across procedures, reinforcing that dynamic modeling better captures surgical impact than cross-sectional endpoints.

Our findings underscore the importance of repeated-measures modeling; endpoint-only analyses may obscure persistent trajectory differences, particularly when procedures are selectively allocated to patients with markedly different baseline BMI profiles.

### 4.2. Muscle Preservation: Proportional Versus Disproportionate Lean Mass Loss

A major concern regarding malabsorptive techniques is excessive lean tissue depletion. Consistent with prior literature [7,8,20,25,26,27,28], our cohort exhibited early reductions in FFM and SMM followed by stabilization after the first postoperative year. Davidson et al. [7] demonstrated similar patterns, with early lean mass decline and a subsequent plateau over 5 years. Although BPD was associated with higher %MML (%MML, 20.6 ± 5.5%, *p* = 0.019), absolute SMM (*p* = 0.388) and appendicular muscle mass (AMM, *p* = 0.194) did not differ significantly between techniques after multivariable adjustment.

This nuance is crucial: body composition modeling literature emphasizes that lean mass loss during weight reduction is strongly driven by baseline adiposity and total weight loss magnitude, reflecting a “proportional remodeling” phenomenon rather than selective tissue degradation [29]. Moreover, mechanistic studies suggest that BS may improve muscle insulin sensitivity and metabolic quality even when lean mass decreases modestly [30]. Improvements in muscle-to-fat ratio and systemic inflammatory status may offset numerical reductions in muscle quantity [27,30].

### 4.3. Sarcopenic Obesity and Diagnostic Variability

Sarcopenic obesity is increasingly recognized among bariatric candidates, with prevalence estimates ranging from 13% to over 20% depending on cut-offs and assessment methods [31,32,33,34]. Importantly, agreement between BIA and DXA-derived thresholds varies substantially [35,36], and reliance on FFM alone may overestimate true functional muscle mass in obesity [11]. Our study did not incorporate functional parameters such as handgrip strength, which are increasingly recommended for sarcopenia diagnosis [30,33]. Therefore, while absolute SMM was preserved longitudinally, future studies integrating functional metrics would strengthen the interpretation of long-term musculoskeletal safety.

### 4.4. Independent Role of Age and Baseline BMI

Age emerged as a robust independent negative predictor of weight and fat mass loss (*p* < 0.001), consistent with previous observational studies linking baseline characteristics to postoperative outcomes [23]. Age-related declines in anabolic responsiveness and resting energy expenditure may attenuate surgical response. Baseline BMI also significantly influenced %WL trajectories (*p* = 0.029), reinforcing the necessity of multivariable adjustment when comparing techniques that are selectively indicated for more severe phenotypes [15,16,17].

### 4.5. Methodological Considerations: Attrition and Longitudinal Modeling

Long-term bariatric cohorts are frequently characterized by substantial attrition [15,20], which reached 72.7% at 60 months in our study. However, sensitivity analyses (Table 2) revealed no significant baseline differences between completers and non-completers, supporting the plausibility of a MAR mechanism. Multivariable LMM provides a robust framework for handling repeated measures under MAR assumptions without restricting analyses to complete cases [22]. Nevertheless, informative missingness cannot be entirely excluded, and the absence of significant inter-technique differences at the isolated 60-month endpoint may partly reflect reduced statistical power in the final sample (*n* = 36).

### 4.6. Body Composition Assessment: BIA in Context

Multi-frequency BIA was used for longitudinal monitoring. Although practical and validated in bariatric populations [9,10,12,26], systematic differences between BIA and DXA have been documented, particularly for regional lean mass assessment [35,36]. Standardized pre-assessment conditions were strictly implemented to minimize hydration-related bias. Nonetheless, incorporation of DXA or imaging modalities in future prospective cohorts would enhance precision.

### 4.7. Limitations

Several limitations warrant consideration. First, the retrospective design precludes causal inference. Second, technique allocation was based on clinical phenotype, introducing potential residual confounding despite multivariable adjustment. Third, high attrition at 5 years limits external validity. Fourth, BIA rather than DXA was used. Fifth, dietary protein intake, supplementation adherence, and physical activity were not systematically assessed, which are known to influence lean mass preservation [14,18].

## 5. Conclusions

After rigorous adjustment for baseline heterogeneity, BPD provides a superior long-term trajectory of adiposity reduction compared with RYGB and SG, without evidence of disproportionate impairment of absolute skeletal muscle mass over a 5-year horizon. These findings reinforce the concept of proportional body composition remodeling following bariatric surgery and highlight the importance of longitudinal modeling and multivariable adjustment in evaluating long-term metabolic outcomes.

## Figures and Tables

**Table 1:** Baseline clinical characteristics of the study population.

	BPD (*n* = 55)	RYGB (*n* = 31)	SG (*n* = 42)	*p*-Value
**Age (years)**	43.1 ± 9.3	46.8 ± 9.1	48 ± 9.4	0.029
**Female (%)**	43.6% (24/55)	29% (9/31)	23.8% (10/42)	0.101
**Weight (kg)**	148.4 ± 28.1	119.8 ± 17.7	113.9 ± 15.6	<0.001
**BMI (kg/m^2^)**	52.3 ± 8.3	43.6 ± 5.8	42.7 ± 4.2	<0.001
**FM (kg)/(%)**	70.9 ± 15.8/47.6 ± 4.1	53.5 ± 11.4/44.5 ± 5.4	49.9 ± 7.7/43.8 ± 3.9	<0.001
**FFM (kg)/(%)**	76.8 ± 14.7/51.9 ± 5.1	65.8 ± 10.5/55.1 ± 5.2	63.0 ± 11.4/55.1 ± 5.3	<0.001
**MM (kg)**	73.7 ± 13.8	63.1 ± 10.3	60.8 ± 9.9	<0.001
**SMM (kg)**	28.7(22.1–34.1)	24.1(21.3–28.6)	23.1(20–26.6)	<0.001
**AMM (kg)**	34.8 (25.5–44.1)	28.2 (23.5–33.8)	24.4 (21.7–29.2)	<0.001

Values are expressed as mean ± standard deviation. BPD: Biliopancreatic Diversion; RYGB: Roux-en-Y Gastric Bypass; SG: Sleeve Gastrectomy; BMI: Body Mass Index. *p*-values derived from one-way ANOVA (continuous variables) and Chi-square test (categorical variables).

**Table 2:** Sensitivity analysis: Comparison between Completers and Non-completers.

	Completers (*n* = 36)	Non-Completers (*n* = 92)	*p*-Value
**Age (years)**	44.3 ± 10.1	45.6 ± 9.4	0.487
**Baseline BMI (kg/m^2^)**	46.5 ± 7.0	47.3 ± 8.4	0.626
**Initial weight (kg)**	125.4 ± 25.5	132.1 ± 27.9	0.208
**Sex (female, %)**	63.9%	67.4%	0.866
**Technique (BPD/RYGB/SG)**	13/11/12	42/20/30	0.501

Values are expressed as mean ± standard deviation at baseline. Completers are defined as patients with a minimum of 60 months of follow-up. *p*-values derived from independent samples *t*-test.

**Table 3:** Multivariable LMM Coefficients for %WL and %FML Trajectories.

Predictor	Beta (%WL) [95% CI]	*p*-Value	Beta (%FML) [95% CI]	*p*-Value
**BPD × Time interaction**	0.124 [0.013, 0.235]	0.028	0.288 [0.135, 0.440]	<0.001
**RYGB × Time interaction**	−0.058 [−0.175, 0.059]	0.329	−0.079 [−0.239, 0.081]	0.333
**Baseline BMI (kg/m^2^)**	0.289 [0.029, 0.548]	0.029	0.117 [−0.301, 0.534]	0.584
**Age (years)**	−0.420 [−0.601, −0.240]	<0.001	−0.630 [−0.920, −0.339]	<0.001
**Sex (Male)**	−0.239 [−3.717, 3.239]	0.893	4.244 [−1.348, 9.837]	0.137

Beta represents the fixed-effect coefficient from the Linear Mixed-Effects Model (LMM). %WL: percentage of total weight loss; %FML: percentage of fat mass loss. Models were adjusted for sex as a covariate. Baseline BMI and Age refer to pre-surgical values. Reference category for technique: Sleeve Gastrectomy.

**Table 4:** Longitudinal Evolution of Anthropometric and Fat Mass Parameters.

Variable	Technique	12 m	24 m	36 m	48 m	60 m	*p*-Value ^1^
**Weight (kg)**	BPD	100.4 ± 22.4	93.4 ± 19.2	93.7 ± 19.2	93.7 ± 20.6	88.8 ± 19.4	<0.001
	RYGB	78.6 ± 14.2	80.2 ± 15.2	83.9 ± 14.2	85.4 ± 14.8	84.8 ± 11.1	
	SG	80.7 ± 16.7	82.1 ± 20.9	80.8 ± 14.1	80.9 ± 14.7	82.2 ± 14.7	
**BMI (kg/m^2^)**	BPD	35.5 ± 7.1	33.4 ± 6.4	33.5 ± 5.9	33.7 ± 6.1	32.3 ± 5.6	<0.001
	RYGB	29.1 ± 5.6	29.5 ± 5.8	30.9 ± 5.5	32.2 ± 5.9	31.2 ± 3.8	
	SG	29.7 ± 3.9	29.4 ± 3.4	29.5 ± 4.4	30.8 ± 3.5	30.9 ± 3.5	
**%WL (%)**	BPD	32.1 ± 10.8	36.1 ± 9.1	37.5 ± 11.4	35.1 ± 14.1	38.5 ± 11.1	0.028
	RYGB	33.1 ± 8.2	31.8 ± 9.1	28.6 ± 8.4	27.7 ± 9.8	25.5 ± 7.8	
	SG	30.0 ± 9.3	27.7 ± 16.1	29.5 ± 9.9	27.7 ± 9.3	27.3 ± 8.2	
**FM (kg)**	BPD	34.5 ± 14.2	30.5 ± 12.1	31.2 ± 12.3	31.5 ± 13.5	28.9 ± 14.2	<0.001
	RYGB	23.5 ± 10.1	25.2 ± 10.4	31.3 ± 12.5	30.9 ± 10.3	28.6 ± 6.1	
	SG	25.1 ± 7.4	25.2 ± 5.8	26.5 ± 6.3	28.5 ± 6.2	29.4 ± 6.6	
**%FML(%)**	BPD	51.2 ± 17.6	56.4 ± 17.2	56.9 ± 18.2	53.8 ± 21.7	57.9 ± 18.3	0.011
	RYGB	55.7 ± 14.1	52.1 ± 14.6	39.3 ± 20.9	41.6 ± 15.3	39.8 ± 14.3	
	SG	50.1 ± 12.6	48.7 ± 12.1	47.2 ± 14.7	42.6 ± 13.8	42.0 ± 12.5	

^1^ *p*-values in this table refer to the overall longitudinal effect of ‘Time’ within the adjusted LMM. Comparative analysis between surgical techniques is reported via the coefficients in Table 3.

**Table 5:** Longitudinal Evolution of Muscle Mass and Fat-Free Mass Parameters.

Variable	Technique	12 m	24 m	36 m	48 m	60 m	*p*-Value ^1^
**FFM (kg)**	BPD	65.9 ± 11.4	63.1 ± 10.2	61.4 ± 12.1	62.2 ± 13.4	62.6 ± 15.1	0.499
	RYGB	55.1 ± 8.7	56.4 ± 10.1	55.8 ± 8.8	54.5 ± 8.7	56.1 ± 9.3	
	SG	55.6 ± 11.2	55.0 ± 9.4	54.3 ± 10.2	52.3 ± 10.4	52.8 ± 10.5	
**%FFML(%)**	BPD	13.8 (10.5–20.2)	18.1 (13.0–21.9)	19.3 (16.1–23.9)	16.9 (12.1–23.8)	20.5 (13.5–23.8)	0.606
	RYGB	14.2 (11.6–17.7)	14.9 (11.2–18.4)	15.1 (11.3–17.5)	16.1 (12.4–19.2)	15.7 (11.4–16.9)	
	SG	15.4 (10.9–18.3)	16.1 (11.9–18.1)	14.7 (11.4–17.1)	14.7 (11.9–17.6)	13.7 (11.4–16.8)	
**MM (kg)**	BPD	62.5 ± 11.1	59.8 ± 10.1	60.0 ± 10.2	59.1 ± 12.4	56.9 ± 11.4	0.043
	RYGB	52.2 ± 8.3	52.5 ± 8.6	53.1 ± 8.4	51.7 ± 8.3	53.3 ± 8.8	
	SG	52.8 ± 10.4	50.8 ± 9.1	51.5 ± 9.8	49.7 ± 10.1	50.1 ± 9.8	
**%MML(%)**	BPD	14.9 ± 6.5	17.8 ± 5.7	18.3 ± 8.3	17.4 ± 10.2	20.6 ± 5.5	0.019
	RYGB	15.9 ± 5.1	15.9 ± 5.1	15.6 ± 5.4	16.8 ± 6.2	15.3 ± 3.8	
	SG	14.2 ± 7.5	16.2 ± 4.4	15.6 ± 6.3	15.7 ± 5.4	15.5 ± 5.3	
**SMM (kg)**	BPD	27.6 (21.3–33.5)	25.7 (21.2–31.9)	25.5 (22.8–32.2)	25.8 (20.3–32.1)	24.8 (21.7–32.9)	0.088
	RYGB	22.1 (19.4–28.2)	21.8 (20.2–25.8)	21.0 (20.1–27.5)	20.5 (19.2–23.4)	22.1 (20.2–27.1)	
	SG	21.7 (18.6–28.6)	20.7 (18.1–25.6)	21.1 (18.5–25.6)	18.7 (17.5–22.4)	19.4 (17.7–23.3)	

^1^ *p*-values in this table refer to the overall longitudinal effect of ‘Time’ within the adjusted LMM. Comparative analysis between surgical techniques is reported via the coefficients in Table 3.

## Data Availability

The data presented in this study are available on request from the corresponding author as per European legislation on data protection.
